#  Lexis Expansion: a prerequisite for analyzing time changing variables in a cohort study 

**DOI:** 10.3126/nje.v7i2.17974

**Published:** 2017-06-30

**Authors:** Nitika, Sidharth Sekhar Mishra, Pallavi Lohani

**Affiliations:** 1 Senior Resident, School of Public Health, PGIMER Chandigarh, India; 2 Faculty, DAMS.,; 3 Junior Resident, Community Medicine, PMCH Patna

**Keywords:** Lexis Expansion, variable, cohort study.

## Abstract

In a prospective analytical cohort study or a study involving a longer follow up, changing age of participants influence the relationship between exposure and outcome. Usually age at entry is incorporated in the regression model to adjust for age. However, this fails to fully explain and adjust for changing age. For this Lexis expansion, a concept by Wilhelm Lexis, allows the analyst to expand the observations as per age bands and check for confounding and interaction by changing age. Lexis expansion assumes the rates to be constant within an age band.

## Introduction

In a prospective analytical cohort study or a study involving a longer follow -up, the characteristics of the individual variable may either change with time (time changing variables) or may remain fixed (fixed variable). One of the examples of a fixed variable is gender. The change in a time- changing variable can be either random or deterministic. The example of the random time changing variable is level of physical activity.

The individual may change from being physically active to being physically inactive or vice-versa. The example of the deterministic time changing variable is age of subjects. With each passing year, the current age of subjects keeps on changing, howsoever the change can be determined or estimated. This is important because age is one of the important risk factors for varied diseases and also for mortality as explained in the example given below.

**Figure 1:**
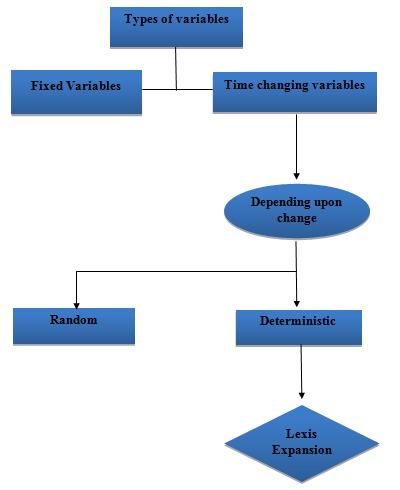
Flowchart showing types of variables and use of Lexis expansion

Lexis expansion is done for time changing variable wherein the change is deterministic [ 1 ] (Figure 1).

In this article, we will first proceed with explaining the concept, then steps of Lexis expansion in Stata followed by using the expanded data for classical analysis and Poisson regression.

### Lexis Expansion- the basic concept:

Lexis expansion expands the data by converting one observation per subject to one observation for each time interval per subject. This is named after Wilhelm Lexis, a German statistician. [ 2 ]This method is also referred to as ' episode splitting method ' . 

 Lets understand this with the help of examples. 

 Example 1 -

**Figure 2:**
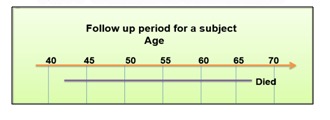
Lexis expansion for a single participant

Suppose a subject enters the study at the age of 42 years and is followed for 25 years. Considering age as a risk factor for most of the diseases, exposure is age more than 60 years. If we divide the follow up duration in two parts, first part being up to the age of 60 years and second part is beyond 60 years. Thus, this subject is changing the status from being unexposed to exposed. We just divided the follow up duration in two parts. Similarly, we can divide the follow up duration into five year intervals of 40-44, 45-49, 50-54, 55-59, 60-64, and 65-69 years(Figure 2).

This method of breaking/ dividing/ splitting the follow up duration into intervals, say 5-years interval is known as Lexis expansion. Thus, with lexis expansion, we can split the entire follow-up duration into age-specific intervals. For each age-specific interval, we have information about a number of events and follow-up duration for both single subject and entire sample/study population. This information is used for calculating age-specific rate. Example 2 –We will consider two subjects (details given in table 1) and expand the data considering age intervals/ age-bands of 25-29, 30-34, 35-39, 40-44, and 45-49 years. (Table 2 )

In Table 2, "D" represents the total number of events and "Y" represents number of person years for a particular interval.

Here, two subjects are used for illustration to understand the concept, otherwise in practice, it can be done for all the subjects in the study.The lexis expansion expands the data, meaning the number of records/row increases. The increase depends upon the number of splits for the follow up duration.

### Analysis in Statistical Software:

Steps in STATA:

Here we are explaining the steps of doing Lexis expansion in Stata. [ 3 ]

1. Declare the data to be survival time data using drop down menu – Statistics < Survival analysis < Set up and utilities < Declare data to be survival-time data.
2. Create age-specific intervals / age-bands to expand the data. For this, use drop down menu - Statistics < Survival analysis < Set up and utilities < Split time span record.

### Practical Use of Lexis Expansion:

"Checking confounders and interactions and interaction by changing age"

Consider that we want to study the effect of smoking on the mortality. For this association, we want to check how the changing age of participants is behaving – either as a confounder or an effect modifier. We can do it in classical analysis (stratification) and in Poisson regression as well.

For classical analysis, proceed as per the following steps:

1. Lexis expansion of the data using the steps explained above
2. Calculation of rate ratios for the relation between mortality and smoking in strata defined by each age-band
3. Assessment of homogeneity across strata. If the rate ratios are similar, then interaction is not present. Adjusted estimate is compared with the crude estimate to assess the confounding
4. If the rate ratios across strata are different – not homogeneous, it is taken as an evidence of interaction and instead of explaining the adjusted estimate, interaction is explained

For Poisson regression, proceed as per the following steps:

1. Lexis expansion of the data using the steps explained above
2. Age band along with smoking is incorporated as independent variable in the Poisson regression model, and mortality as a dependent variable

## Conclusion

In a long term follow up study, age is one of the important predictor variables. One way of adjusting for this is incorporating age at entry in study into the model. However, this does not take into account the effect of changing age, i.e. current age. The Lexis expansion allows the analyst to adjust for deterministic type of time changing variable (for example, current age and calendar years) in a classical analysis and in Poisson regression as well. However, for Lexis expansion, we assume that the rates remain constant in each age-specific interval, i.e. it is not changing within an interval. If it is changing rapidly, then cox regression is a better technique to handle this.

## Figures and Tables

**Table 1:** Table showing the follow up duration and profile of participant in the study

Subject	Date of Birth	Date of entry	Age at entry	End of follow up	Age at exit	Outcome
1	1/3/1956	1/5/1981	25.2	1/8/1998	42.5	Alive
2	01/4/1956	1/10/1983	27.6	1/6/2004	48.2	Dead

**Table 2:** Lexis expansion for two participants with number of events and total follow up duration in each age-specific

Age bands		25-29	30-34	35-39	40-44	45-49
**Subject 1**	D	0	0	0	0	0
	Y	4.8	5	5	2.5	0
**Subject 2**	D	0	0	0	0	1
	Y	2.4	5	5	5	3.2
**Total **	D	0	0	0	0	1
	Y	7.2	10	10	7.5	3.2

## References

[1] Rayco-Solon P Moore SE Fulford AJ Prentice AM. Fifty-year mortality trends in three rural African villages. Tropical medicine & international health.2004 Nov 1; 9 (11): 1151 - 60. https://doi.org/10.1111/j.1365-3156.2004.01325.x PMid: 15548310 10.1111/j.1365-3156.2004.01325.x

[2] Hertz S Wilhelm Lexis. In:C.C. Heyde, E. Seneta, P. Crepel, S.E. Fienberg, J. Gani eds.. Statisticians of the Centuries.1st ed. New York: Springer, 2001 ,pp 204 - 207. https://doi.org/10.1007/978-1-4613-0179-0_43

[3] Jenkins SP, Survival Analysis with STATA. [online] 2008 [cited 2017 June 06]. Available from: URL: https://www.iser.essex.ac.uk/resources/survival-analysis-with-stata

